# Predictive modeling and risk assessment of pancreatic β-cell dysfunction in type 2 diabetes

**DOI:** 10.1097/MD.0000000000044462

**Published:** 2025-09-19

**Authors:** Nan Wu, Yuqin Sun

**Affiliations:** a General Practice, North China University Affiliated Hospital, Jilin, Jilin Province, China; b Department of Cardiovascular, North China University Affiliated Hospital, Jilin, Jilin Province, China.

**Keywords:** influencing factors, insulin resistance, pancreatic β cell dysfunction, type 2 diabetes mellitus

## Abstract

Type 2 diabetes mellitus (T2DM) is a widespread metabolic disorder driven in part by pancreatic β-cell dysfunction. This study aims to identify key determinants of β-cell impairment in T2DM patients and develop a predictive risk model to enhance clinical management strategies. This retrospective study included 120 patients newly diagnosed with T2DM who were admitted to our hospital between March 2023 and March 2024. We measured fasting plasma glucose, fasting insulin, and other relevant metabolic indicators. Insulin resistance and β-cell function were evaluated using the homeostasis model assessment (homeostasis model assessment insulin-resistance and homeostasis model assessment-β). To identify independent risk factors, we performed both univariate and multivariate stepwise regression analyses, and subsequently constructed predictive models based on the results. The cohort exhibited moderate insulin resistance (homeostasis model assessment insulin-resistance: 2.93 ± 0.77) and significant β-cell dysfunction (homeostasis model assessment-β: 33.74 ± 11.46). Regression analysis identified age, body mass index (BMI), sedentary lifestyle, and triglyceride levels as independent predictors of insulin resistance (*P* < .05), yielding the following model: logit (1) = 2.371 + 0.862 × age + 0.612 × BMI + 0.598 × sedentary + 0.237 × triglyceride. Similarly, age, BMI, and sedentary behavior were independently associated with β-cell dysfunction (*P* < .05), leading to the model: logit (2) = 29.562 + 2.947 × age + 9.570 × BMI + 3.205 × sedentary. Newly diagnosed T2DM patients frequently present with insulin resistance and β-cell impairment. High-risk subgroups include individuals aged ≥ 40, those with elevated BMI, physical inactivity, and dyslipidemia. The proposed predictive models may aid in early risk stratification and personalized intervention.

## 1. Introduction

Diabetes mellitus is a chronic metabolic disorder characterized by elevated blood glucose levels resulting from insufficient insulin secretion or impaired insulin action. Clinically common, the primary type is type 2 diabetes mellitus (T2DM), accounting for 90% of cases.^[1]^ According to the International Diabetes Mellitus, there were 463 million MD cases globally in 2019 (9.3%), with projections for 2030 and 2045 reaching 578 million (10.2%) and 700 million (10.9%), respectively. This upward trend is primarily attributable to global population aging, rising economic prosperity, and accelerated urbanization.^[2]^ Recent research denotes a trend towards a younger onset of T2DM.^[3,4]^ The occurrence and progression of T2DM can easily lead to microvascular and macrovascular complications, culminating in adverse outcomes, making T2DM a serious public health issue both globally and in China.^[5]^

Since T2DM is a chronic progressive disease, it is primarily characterized by insulin resistance and pancreatic β cell dysfunction.^[6]^ Insulin resistance is not exclusively a pathological condition; it may also arise in physiological states such as puberty and late pregnancy.^[7]^ Essentially, it is a compensatory mechanism of the body for excess energy, primarily enhancing glucose uptake in skeletal muscles, myocardium, and adipose tissues, and suppressing glycogenolysis and gluconeogenesis in the liver. When these functions diminish, it indicates the presence of insulin resistance.^[8]^ The latter manifests in various forms: early insulin secretion deficiency, first-phase insulin secretion deficiency, abnormalities in the conversion of proinsulin to insulin, and alterations in insulin secretion patterns.^[9,10]^ As T2DM progresses naturally, it typically shows progressively elevated blood glucose levels, progressively reduced insulin secretion, and persistent insulin resistance. These factors can interact, creating a vicious cycle that further exacerbates T2DM.^[11]^ Additionally, the levels of insulin resistance and pancreatic β cell dysfunction in newly diagnosed T2DM patients are heterogeneous, with varying influencing factors. Accurate early diagnosis and assessment of insulin resistance and pancreatic β cell dysfunction levels in T2DM patients, along with identification of independent risk factors, can facilitate proactive measures to curb T2DM progression.

Traditionally, the glucose clamp technique has been the gold standard for assessing insulin resistance and pancreatic β cell dysfunction. Nevertheless, this approach is invasive, technically challenging, time-consuming, and thus not easily implemented in widespread clinical practice.^[12]^ This study employs the homeostasis model assessment insulin-resistance (HOMA-IR) and homeostasis model assessment-β (HOMA-β) indices, which are simple, noninvasive, and broadly applicable, to evaluate insulin resistance and pancreatic β cell dysfunction. Furthermore, this investigation probes into independent risk factors to provide guidance for personalized diagnosis and treatment of newly diagnosed T2DM patients.

## 2. Materials and methods

### 2.1. General data

This study was approved by the Ethics Committee of North China University Affiliated Hospital. A retrospective study was implemented on 120 newly diagnosed individuals with T2DM admitted to our medical facility from March 2023 to March 2024.

*Inclusion criteria*: compliance with the standards set by the American Diabetes Association in the guidelines stipulated in *Standards of Medical Care in Diabetes*^[13]^; all patients were newly diagnosed with T2DM; patients and their families provided informed consent for the study.

*Exclusion criteria*: type 1 diabetes or gestational diabetes mellitus (GDM); presence of diabetes complications; coexistence of malignant tumors; recent use of medications affecting blood glucose and blood lipid levels. The research was approved by the hospital’s ethics committee.

### 2.2. Methods

#### 2.2.1. Demographic information

Trained medical personnel were selected to administer a self-designed demographic survey questionnaire. This questionnaire collected demographic information including gender (male/female), age (≤40 years/>40 years), body mass index (BMI) [overweight and obese (≥24 kg/m²), normal and underweight (<24 kg/m²)], family history of diabetes (presence/absence), smoking history (presence/absence), alcohol consumption history (presence/absence), and sedentary behavior (defined as sitting for ≥ 6 hours a day as “presence” otherwise “absence”).

#### 2.2.2. Clinical blood collection

On the day following enrollment, 5 mL of peripheral venous blood was harvested from all participants in a fasting state. Using the AU 5800 automated biochemical analyzer (Beckman Coulter, Brea), the following parameters were gauged: fasting plasma glucose (FPG) (normal range 3.9–6.1 mmol/L), fasting insulin (FINS) (normal range 10–25 mmol/L), triglycerides (TGs) (normal range 0.45–1.69 mmol/L), total cholesterol (normal range 3.0–5.7 mmol/L), high-density lipoprotein cholesterol (normal range 1.16–1.55 mmol/L), and low-density lipoprotein cholesterol (normal range < 3.4 mmol/L).

### 2.3. Observation of indicators

The insulin resistance and pancreatic β cell dysfunction of all participants were observed and assessed using HOMA-IR and HOMA-β, respectively. HOMA-IR was calculated as per the formula: HOMA-IR = FPG × FINS/22.5. HOMA-β was calculated using the formula: HOMA-β = 20 × FINS/(FPG − 3.5).

### 2.4. Sample size and power analysis

Prior to data analysis, a post hoc power analysis was conducted to assess whether the sample size of 120 patients was adequate for the multivariate regression models used in this study. Assuming a medium effect size (*f*² = 0.15), a significance level (α) of 0.05, and 4 predictors included in the final regression model, the minimum required sample size for achieving 80% statistical power was calculated to be approximately 85 cases, based on G*Power 3.1 software (Heinrich-Heine-Universität Düsseldorf, Düsseldorf, North Rhine-Westphalia , Germany). Therefore, the sample size of 120 participants in this study exceeded the minimum requirement and was considered sufficient to support the stability and reliability of the regression analysis results. Nonetheless, we acknowledge that larger multicenter samples may further improve the generalizability of our findings.

### 2.5. Statistical analysis

The data was processed and analyzed via SPSS 22.0 software (IBM, Armonk). Normally distributed measurement data were displayed as “mean ± SD” and analyzed through *t* tests. Enumeration data were exhibited as “%” and analyzed via *χ*² tests. Univariate and multivariate stepwise linear regression models were employed to identify independent risk factors influencing insulin resistance and pancreatic β cell dysfunction in T2DM patients. A *P*-value of <.05 contained statistical significance.

## 3. Results

### 3.1. Assessment of insulin resistance and pancreatic β cell dysfunction in newly diagnosed T2DM patients

**Figure 1.**
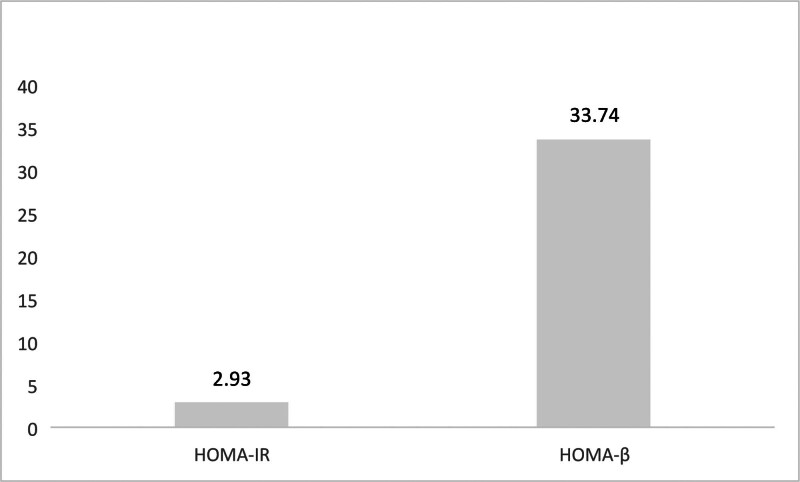
Assessment of insulin resistance and pancreatic β cell dysfunction in newly diagnosed T2DM patients. T2DM = type 2 diabetes mellitus.

Among the 120 T2DM cases, the HOMA-IR value reflecting insulin resistance reached (2.93 ± 0.77), whereas the HOMA-β value signifying pancreatic β cell dysfunction stood at (33.74 ± 11.46). For details, refer to Figure 1.

### 3.2. Univariate analysis of insulin resistance in newly diagnosed T2DM patients

Demographic and clinical data were stratified, and the outcomes of the univariate analysis unraveled the following: T2DM patients aged ≥ 40 years had a HOMA-IR of (3.53 ± 0.62), which was higher than the (2.47 ± 0.53) observed in patients younger than 40 years. Overweight and obese T2DM patients exhibited a HOMA-IR of (3.34 ± 0.68), surpassing the (2.42 ± 0.56) seen in normal weight and underweight patients. Sedentary T2DM patients displayed a HOMA-IR of (3.33 ± 0.68), compared to (2.41 ± 0.55) in non-sedentary patients. T2DM patients with abnormal TG levels had a HOMA-IR of (3.16 ± 0.74), higher than the (2.40 ± 0.57) in those with normal TG levels. All differences were statistically significant (*P* < .05), as revealed in Table 1.

**Table 1:** Univariate analysis of insulin resistance in newly diagnosed T2MD patients.

Items		Number of cases (n = 120)	HOMA-IR	*t*	*P*
Gender	Male	75	2.92 ± 0.72	0.151	.880
	Female	45	2.95 ± 0.87		
Age	≥40 years	52	3.53 ± 0.62	10.013	.000
	<40 years	68	2.47 ± 0.53		
BMI	Overweight and obese	67	3.34 ± 0.68	7.930	.000
	Normal weight and underweight	53	2.42 ± 0.56		
Family history of diabetes	Presence	24	3.14 ± 0.50	1.512	.133
	Absence	96	2.88 ± 0.82		
Smoking history	Presence	42	2.98 ± 0.85	0.591	.556
	Absence	78	2.90 ± 0.72		
Drinking history	Presence	49	2.89 ± 0.96	0.467	.641
	Absence	71	2.96 ± 0.66		
Sedentary behavior	Presence	68	3.33 ± 0.68	7.910	.000
	Absence	52	2.41 ± 0.55		
TG	Abnormal	84	3.16 ± 0.74	5.483	.000
	Normal	36	2.40 ± 0.57		
TC	Abnormal	78	2.99 ± 0.85	1.060	.291
	Normal	42	2.83 ± 0.61		
HDL-C	Abnormal	73	2.97 ± 0.70	0.752	.453
	Normal	47	2.86 ± 0.88		
LDL-C	Abnormal	70	2.98 ± 0.82	0.765	.446
	Normal	50	2.87 ± 0.71		

BMI = body mass index, HDL-C = high-density lipoprotein cholesterol, HOMA-IR = homeostasis model assessment insulin-resistance, LDL-C = low-density lipoprotein cholesterol, T2DM = type 2 diabetes mellitus, TG = triglyceride.

### 3.3. Multivariate stepwise linear regression analysis of insulin resistance in newly diagnosed T2DM patients

The outcomes obtained from the multivariate stepwise linear regression analysis confirmed that age, BMI, sedentary behavior, and TG levels were all independent risk factors influencing insulin resistance in newly diagnosed T2DM patients (*P*_all_ < 0.05). With *R*² and adjusted *R*² values approaching 1, the risk model displayed a good fit. The risk model was established based on HOMA-IR (predicted) = 2.371 + 0.862 × age + 0.612 × BMI + 0.598 × sedentary + 0.237 × TG. See Table 2.

**Table 2:** Multivariate stepwise linear regression analysis of insulin resistance in newly diagnosed T2MD patients.

Variables	B	SE	Beta	*t*	*P*	95% CI
Constants	2.371	0.095	/	24.824	.000	2.181–2.560
Age	0.862	0.167	0.554	5.148	.000	0.530–1.194
BMI	0.612	0.124	0.368	4.016	.002	0.363–0.860
Sedentary behavior	0.598	0.117	0.356	4.023	.003	0.362–0.833
TG	0.237	0.113	0.147	2.097	.038	0.013–0.461
*R* ^*2*^			0.927			
Adjusted *R*^*2*^			0.927			

BMI = body mass index, TG = triglyceride, T2DM = type 2 diabetes mellitus.

### 3.4. Univariate analysis of pancreatic β cell dysfunction in newly diagnosed T2DM patients

Demographic and clinical data were stratified, and the findings of the univariate analysis unveiled that T2DM patients aged ≥ 40 years exhibited a HOMA-β of (36.38 ± 9.65), which was higher than the (31.72 ± 12.44) observed in patients younger than 40 years. Overweight and obese T2DM patients displayed a HOMA-β of (37.05 ± 10.48), surpassing the (29.57 ± 11.48) seen in normal weight and underweight individuals. Sedentary T2DM patients displayed a HOMA-β of (36.84 ± 10.33), higher than the (29.69 ± 11.79) in non-sedentary patients. All variances were considered statistically significant (*P* < .05). Refer to Table 3.

**Table 3:** Univariate analysis of pancreatic β cell dysfunction in newly diagnosed T2MD patients.

Items		Number of cases (n = 120)	HOMA-β	*t*	*P*
Gender	Male	75	33.43 ± 11.21	0.386	.700
	Female	45	33.27 ± 12.09		
Age	≥40 years	52	36.38 ± 9.65	2.236	.027
	<40 years	68	31.72 ± 12.44		
BMI	Overweight and obese	67	37.05 ± 10.48	3.721	.000
	Normal weight and underweight	53	29.57 ± 11.48		
Family history of diabetes	Presence	24	36.98 ± 9.40	1.549	.124
	Absence	96	32.94 ± 11.88		
Smoking history	Presence	42	35.82 ± 11.91	1.656	.100
	Absence	78	32.31 ± 11.07		
Drinking history	Presence	49	34.97 ± 11.91	0.856	.393
	Absence	71	33.08 ± 11.30		
Sedentary behavior	Presence	68	36.84 ± 10.33	3.535	.001
	Absence	52	29.69 ± 11.79		
TG	Abnormal	84	34.95 ± 10.81	1.756	.082
	Normal	36	30.95 ± 12.70		
TC	Abnormal	78	34.29 ± 11.28	0.708	.481
	Normal	42	32.73 ± 11.99		
HDL-C	Abnormal	73	34.13 ± 11.53	0.458	.648
	Normal	47	33.14 ± 11.56		
LDL-C	Abnormal	70	34.09 ± 11.99	0.388	.699
	Normal	50	33.26 ± 10.89		

BMI = body mass index, HDL-C = high-density lipoprotein cholesterol, HOMA-β = homeostasis model assessment-β, LDL-C = low-density lipoprotein cholesterol, TG = triglyceride.

### 3.5. Multivariate stepwise linear regression analysis of pancreatic β cell dysfunction in newly diagnosed T2DM patients

Multivariate stepwise linear regression analysis demonstrated that age, BMI, and sedentary behavior were all independent risk factors affecting pancreatic β cell dysfunction in newly diagnosed T2DM patients (*P*_all_ < 0.05). The model fit was good with *R*^2^ and adjusted *R*^2^ approaching 1. The risk model was established as Logit (2) = 29.562 + 2.947 × age + 9.570 × BMI + 3.205 × sedentary, as shown in Table 4.

**Table 4:** Multivariate stepwise linear regression analysis of pancreatic β cell dysfunction in newly diagnosed T2MD patients.

Variables	B	SE	Beta	*t*	*P*	95% CI
Constants	29.562	1.528	/	19.344	.000	21.457–34.343
Age	2.947	1.013	2.513	1.589	.048	0.920–4.972
BMI	9.570	2.436	8.354	4.657	.000	4.699–14.441
Sedentary behavior	3.205	1.216	2.720	2.002	.022	0.772–5.636
*R* ^*2*^			0.938			
Adjusted *R*^*2*^			0.938			

BMI = body mass index.

## 4. Discussion

The occurrence and development of T2DM are closely associated with insulin resistance and pancreatic β cell dysfunction. Nevertheless, variances in insulin resistance and pancreatic β cell dysfunction levels exist among different individuals with T2DM at the time of initial diagnosis. This study adopted internationally recognized HOMA-IR and HOMA-β to evaluate insulin resistance and pancreatic β cell dysfunction levels. However, The HOMA-IR index is influenced by variables including ethnicity, sex, and population characteristics, and there is currently no unified consensus on the optimal cutoff value for insulin resistance. Research on Spanish adolescents has confirmed a HOMA-IR cutoff value of 2.60 for insulin resistance.^[14]^ In a study recruiting 3203 Chinese children (aged 6–18 years), the cutoff value for insulin resistance HOMA-IR was determined to be 3.0.^[15]^ As for HOMA-β, it is 100% in normal individuals, but following the onset of T2DM, if there is a decline in pancreatic β cell function, HOMA-β will decrease accordingly.^[16]^ In accordance with the findings of this research, among the 120 newly diagnosed T2DM patients included, HOMA-IR was (2.93 ± 0.77) and HOMA-β was (33.74 ± 11.46). Based on years of clinical experience in our hospital, abnormal levels of HOMA-IR and HOMA-β are present, denoting varying degrees of insulin resistance and pancreatic β cell dysfunction in newly diagnosed T2DM patients, which require urgent attention.

Moreover, further confirmation of the independent risk factors for insulin resistance and pancreatic β cell dysfunction in newly diagnosed T2DM patients can help provide important references for personalized treatment plans. Considering the effective definition of HOMA-IR and HOMA-β levels, this study employed a multiple stepwise linear regression model for multivariate analysis. Literature search has revealed that factors leading to insulin resistance and pancreatic β cell dysfunction in T2DM are diverse. Research has discovered that T2DM is known as a polygenic hereditary disease, with a cross-sectional study encompassing 814 Maltese-Caucasian individuals confirming that polygenic lipodystrophy risk alleles can drive insulin resistance risk.^[17]^ Based on the prospective cohort study of Dongfeng-Tongji University, it was displayed that BMI is positively correlated with T2DM risk in the elderly Chinese population, and it is more efficacious in forecasting T2DM than other anthropometric parameters (AUC value is 0.655).^[18]^ Based on a long-term follow-up study^[19]^ of 116,855 Chinese adults aged 20 or older, it was demonstrated that during a median follow-up of 2.98 years, 2685 subjects developed T2DM, with an incidence rate of 2.30%. The research also found that TG/high-density lipoprotein cholesterol contributed to the hazard ratio of developing T2DM at 1.177, hinting that abnormal lipids metabolism may augment the risk of T2DM, indirectly bringing about insulin resistance or pancreatic β cell dysfunction. In addition, unhealthy lifestyles such as drinking and smoking may exacerbate insulin resistance or pancreatic β cell dysfunction in T2DM patients.^[20]^ According to the findings of this investigation, univariate and multivariate stepwise linear regression analysis confirmed that the independent risk factors affecting insulin resistance in newly diagnosed T2DM patients encompassed age, BMI, sedentary lifestyle, and TG. As for the independent risk factors for pancreatic β cell dysfunction in newly diagnosed T2DM patients, they were age, BMI, and sedentary lifestyle. This is somewhat similar to the aforementioned research, but the conclusions are different to a certain extent, as it did not confirm that a family history of diabetes may culminate in insulin resistance or pancreatic β cell dysfunction, possibly due to the small sample size of this research, only 120 cases, and further verification with a larger sample size is warranted. This finding has demonstrated that age, BMI, and sedentary lifestyle are the most important factors influencing insulin resistance and pancreatic β cell dysfunction in newly diagnosed T2DM patients, whereas TG mainly exacerbates insulin resistance. A brief analysis is as follows: age: as age increases, glucose tolerance function is abated, leading to insulin secretion disorders and insulin resistance,^[21]^ aligned with previous research^[22]^; BMI: BMI standards for overweight and obese patients can trigger the abnormal accumulation of abdominal adipose tissues, thereby attenuating the body’s sensitivity to insulin and further impairing pancreatic β cell function,^[23]^ consistent with related research conclusions^[24,25]^; sedentary behavior: the specific mechanism by which a sedentary lifestyle affects insulin resistance and pancreatic β cell dysfunction is poorly understood. An explanation may be that long-term muscle disuse can lead to muscle atrophy or fibrotic alterations, thereby aggravating muscle insulin resistance^[26–28]^; TG: long-term abnormal TG levels can compete with glucose, culminating in glucose oxidase utilization disorders, and can release large amounts of free fatty acids to interfere with insulin binding to its receptors, hence abating insulin biological efficacy and aggravating insulin resistance.^[29,30]^ These factors may contribute to T2DM pathogenesis through different mechanisms. HOMA-IR and HOMA-β are widely used surrogate indices to evaluate different aspects of glucose metabolism dysfunction. HOMA-IR primarily reflects insulin resistance, calculated as FPG × FINS/22.5, and is influenced by hepatic insulin sensitivity. A higher HOMA-IR indicates reduced insulin responsiveness and is commonly associated with obesity, sedentary behavior, and dyslipidemia. In contrast, HOMA-β evaluates pancreatic β-cell secretory function using the formula: 20 × FINS/(FPG − 3.5). It estimates the ability of β-cells to compensate for insulin demand. A lower HOMA-β suggests diminished insulin secretion, which is a hallmark of β-cell failure in T2DM progression. Clinically, these 2 indices complement each other: HOMA-IR identifies patients with early insulin resistance, who may benefit from lifestyle or pharmacologic interventions targeting insulin sensitivity (e.g., metformin, TZDs), whereas HOMA-β helps assess residual β-cell capacity, guiding decisions on whether to initiate insulin therapy or incretin-based agents. Understanding both parameters provides a more comprehensive picture of disease pathophysiology and aids in individualized treatment planning. These findings highlight the potential clinical utility of HOMA-IR and HOMA-β as complementary tools in early screening and management of T2DM. However, several limitations of this study should be acknowledged.

### 4.1. Limitations

This study has several limitations that should be acknowledged. First, although we identified significant predictors of insulin resistance and β-cell dysfunction, we were unable to comprehensively control for all potential confounding factors, such as dietary habits, psychosocial stress, sleep quality, and use of medications that may affect glucose metabolism. These variables were not systematically recorded in the retrospective data collection process, which may have introduced residual confounding into our model. Future prospective studies incorporating detailed lifestyle and medication data are warranted to improve the precision and generalizability of the predictive models.

## 5. Conclusion

In conclusion, this study demonstrates that newly diagnosed patients with T2DM commonly present with varying degrees of insulin resistance and pancreatic β-cell dysfunction, particularly among individuals aged 40 years and older, those who are overweight or obese, physically inactive, and those with abnormal lipid profiles. By identifying key independent risk factors (namely age, BMI, sedentary behavior, and triglyceride levels) we developed 2 predictive models that can estimate the likelihood and severity of insulin resistance and β-cell dysfunction. These models are simple, noninvasive, and based on routinely collected clinical indicators, making them highly applicable in primary care and early screening settings. They can assist clinicians in stratifying patient risk profiles, enabling early identification of high-risk individuals and promoting timely interventions such as lifestyle modifications or pharmacologic therapies. Ultimately, this may contribute to better glycemic control, reduced disease progression, and improved long-term outcomes in T2DM management. Further validation in larger, multicenter cohorts is warranted to enhance the robustness and generalizability of these predictive tools.

## Author contributions

**Conceptualization:** Nan Wu, Yuqin Sun.

**Data curation:** Nan Wu, Yuqin Sun.

**Formal analysis:** Nan Wu, Yuqin Sun.

**Investigation:** Nan Wu, Yuqin Sun.

**Methodology:** Nan Wu, Yuqin Sun.

**Supervision:** Nan Wu, Yuqin Sun.

**Validation:** Nan Wu, Yuqin Sun.

**Visualization:** Nan Wu, Yuqin Sun.

**Writing – original draft:** Nan Wu, Yuqin Sun.

**Writing – review & editing:** Nan Wu, Yuqin Sun.
